# Effects of Dietary Protein Source and Quantity during Weight Loss on Appetite, Energy Expenditure, and Cardio-Metabolic Responses

**DOI:** 10.3390/nu8020063

**Published:** 2016-01-26

**Authors:** Jia Li, Cheryl L. H. Armstrong, Wayne W. Campbell

**Affiliations:** Department of Nutrition Science, Purdue University, West Lafayette, IN 47907, USA; li1201@purdue.edu (J.L.); carmstro@iupui.edu (C.L.H.A.)

**Keywords:** high-protein diets, satiety, thermogenesis, metabolic syndrome, weight loss

## Abstract

Higher protein meals increase satiety and the thermic effect of feeding (TEF) in acute settings, but it is unclear whether these effects remain after a person becomes acclimated to energy restriction or a given protein intake. This study assessed the effects of predominant protein source (omnivorous, beef/pork *vs.* lacto-ovo vegetarian, soy/legume) and quantity (10%, 20%, or 30% of energy from protein) on appetite, energy expenditure, and cardio-metabolic indices during energy restriction (ER) in overweight and obese adults. Subjects were randomly assigned to one protein source and then consumed diets with different quantities of protein (4 weeks each) in a randomized crossover manner. Perceived appetite ratings (free-living and in-lab), TEF, and fasting cardio-metabolic indices were assessed at the end of each 4-week period. Protein source and quantity did not affect TEF, hunger, or desire to eat, other than a modestly higher daily composite fullness rating with 30% *vs.* 10% protein diet (*p* = 0.03). While the 20% and 30% protein diets reduced cholesterol, triacylglycerol, and APO-B *vs.* 10% protein (*p* < 0.05), protein source did not affect cardio-metabolic indices. In conclusion, diets varying in protein quantity with either beef/pork or soy/legume as the predominant source have minimal effects on appetite control, energy expenditure and cardio-metabolic risk factors during ER-induced weight loss.

## 1. Introduction

An energy deficit is required for overweight and obese adults to lose weight, and research shows that reducing energy intake and retaining fasting and postprandial resting energy expenditure both support this outcome [1]. Typically, a diet-induced energy deficit is associated with increased hunger or reduced fullness [2,3,4]. Since protein is generally recognized as the most satiety-inducing macronutrient [5] and some acute and short-term feeding studies support that higher protein intake increases satiety and reduces energy intake at the next meal [6,7], adults who are dieting may be encouraged to consume high-protein diets.

Importantly, most of these acute and short-term protein feeding studies were conducted using subjects who were consuming their usual self-chosen, energy balanced diets prior to the testing days. Also, the protein content of the test meals varied greatly among studies and was often outside of the acceptable macronutrient distribution range (AMDR) of 10%–35% of energy from protein [8]. Thus, the subjects did not become acclimated to an energy deficit state or to the protein content of the meals/diets they would habitually consume while dieting. Limited research exists on the impact of dietary protein quantity on postprandial and daily appetitive responses from subjects acclimated to the energy and protein contents of the diets, with inconsistent findings and methodologies used to measure perceived appetite [3,9,10,11,12].

High protein intake is also purported to aid body mass loss by helping preserve resting energy expenditure (REE) and increase the thermic effect of feeding (TEF) during a period of energy restriction. A recent meta-analysis showed that high protein diets mitigate the loss of lean body mass during weight loss [4], which is pivotal for maintaining resting energy expenditure [13]. Also, since protein has the highest TEF among the macronutrients, a mixed meal containing higher protein should promote a negative energy balance [10,14,15]. Collectively, higher protein intake during energy restriction should support body mass loss via both appetitive and energetic effects, but evidence that protein-dependent differences in appetite and thermogenesis occur when adults have become acclimated to the diets is limited.

Previous studies suggest that protein source may also affect appetitive responses and resting energy expenditure (fasting and TEF) and potentially explain the inconsistencies among studies. Some research indicates that protein source does [16,17,18] or does not [19,20] influence satiety and/or subsequent food intake, and does [19,21] or does not [22] influence resting energy expenditure.

The purpose of this study was to assess the effects of dietary protein intakes across the ADMR with beef/pork or soy/legume as the predominant protein source on daily and postprandial appetitive responses in overweight/obese adults who were acclimated to an energy-restricted diet. We hypothesized that increasing protein intake would improve daily and postprandial satiety, while predominant protein sources would not affect these parameters differently. We also assessed the effects of these protein diets on fasting and postprandial thermogenic responses, as well as indices of cardio-metabolic health (secondary aims).

## 2. Materials and Methods

### 2.1. Recruitment and Screening

**Figure 1:**
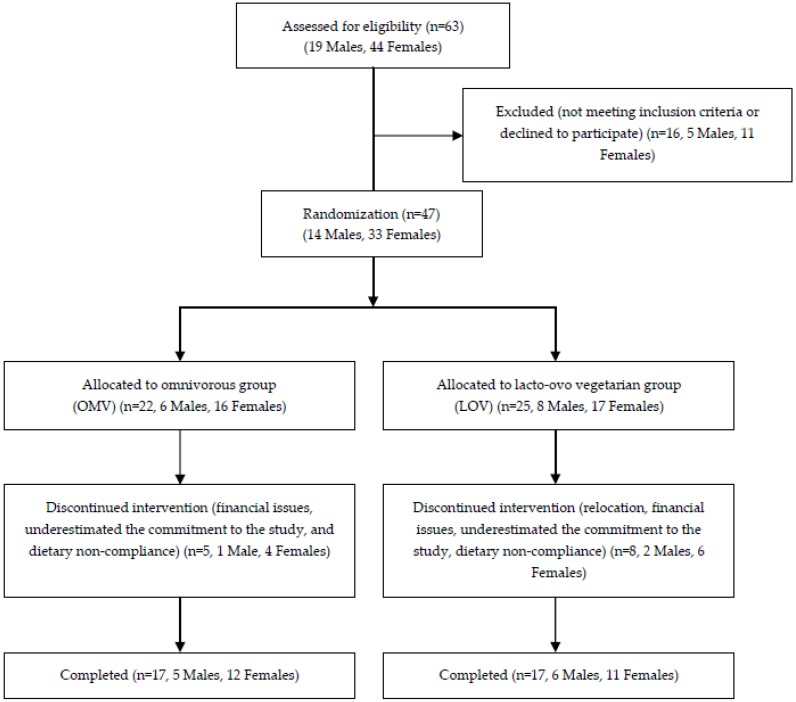
Study recruitment flow diagram.

Subjects were recruited from the greater Lafayette, IN, region using advertisements in newspapers, community bulletin boards, and campus mail. Inclusion criteria were: (1) 21 years and older; (2) body mass index (BMI) range 27.0–36.9 kg/m^2^; (3) non-smoking; (4) weight stable (±3 kg) and stable habitual physical activity patterns during previous 3 months; (5) energy need for weight maintenance 2000–3150 kcal/day; (6) not dietary restrained, ≤14 on hunger scale [23]; (7) post-menopausal, or regularly menstruating women not pregnant or lactating; (8) no acute illness; (9) absence of diabetes mellitus, hypertension, and chronic diseases or use of medications known to influence protein or energy metabolism; (10) blood profile (from the screening visit) within 10% of clinical normalcy (glucose, lipid-lipoprotein profile, liver function enzymes, creatinine); and (11) willingness to eat study foods and able to travel to the testing facility. Prior to enrollment, each subject’s medical history and screening tests were reviewed and medically approved by the study physician. Each subject signed an informed consent document approved by the Purdue University Institutional Review Board and was monetarily reimbursed. Forty-seven adults met the screening requirements and were enrolled. Thirteen subjects (28%) failed to complete the study due to various reasons including relocation, financial issues and dietary non-compliance. Data from 34 subjects were analyzed and reported (30 Caucasian (88%), 1 Asian, 1 African American, 1 Hispanic, 1 other). Detailed numbers regarding subject recruitment, screening, enrollment, randomization, and participation are shown in Figure 1. The study is registered as NCT01005563 at clinicaltrials.gov.

### 2.2. Experimental Design

#### 2.2.1. Study Design Overview

**Figure 2:**
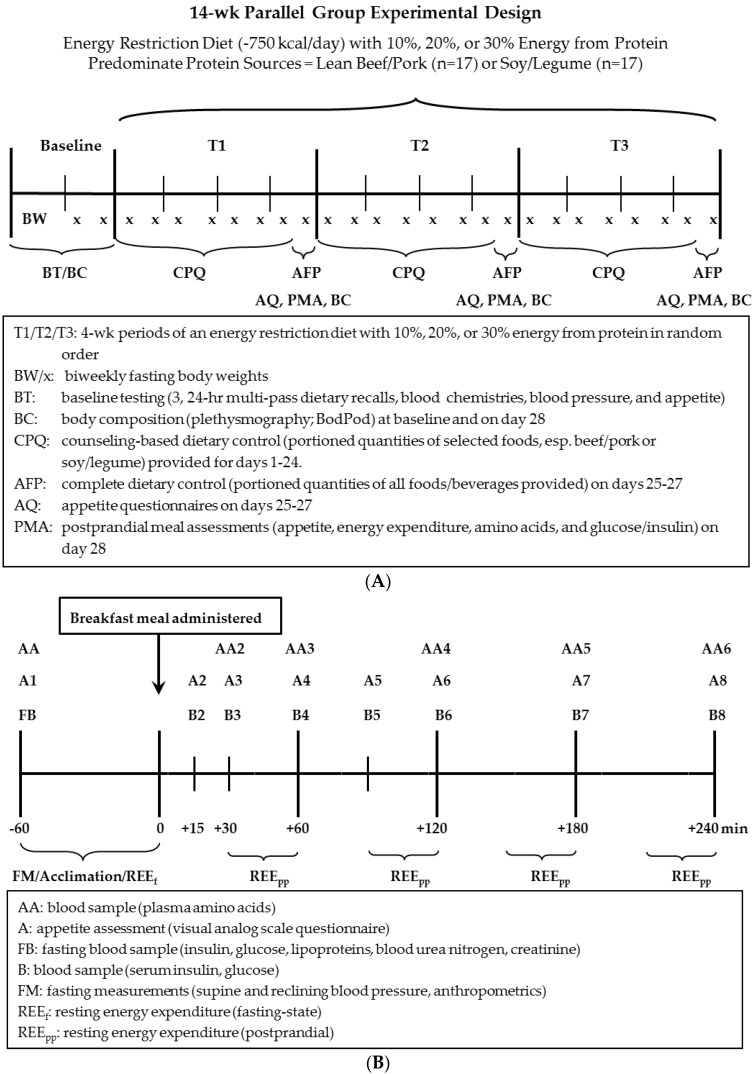
Schematic representation of study design and timeline: (**A**) longitudinal; (**B**) acute study at the end of each 4-week period (day 28).

Following enrollment, subjects completed 2 weeks of baseline data collection and were randomly assigned to either the omnivorous (OMV, beef/pork as predominant protein source) or lacto-ovo vegetarian (LOV, soy/legume as the predominant protein source) group (Figure 2A). During baseline, fasting blood chemistries, blood pressures, body composition/anthropometrics were measured on two separate days, fasting-state energy expenditure on one day, and hourly appetite over three days. Next, subjects in the OMV and LOV groups completed, in random order (crossover design), three consecutive 4-week periods where they consumed an energy-restricted diet (−750 kcal/day) varying in protein quantity (10%, 20%, 30% of energy intake). At the end of each 4-week period, hourly appetite was measured on days 25–27; blood pressures, body composition/anthropometrics, fasting-state and postprandial-state appetite, energy expenditure, glycemic response, and plasma free amino acids (in a subset of 10 subjects) were measured on day 28 (Figure 2B).

#### 2.2.2. Longitudinal Feeding Component of Study (Three Consecutive 4-Week Dietary Intervention Periods)

Diet: Each subject’s energy requirement was estimated using the United States Institute of Medicine’s sex-specific equation for overweight and obese adults [24]. The physical activity factor was 1.12 for men and 1.16 for women. The −750 kcal/day energy-restricted diets contained 10%, 20%, or 30% energy from protein, 25% energy from fat, and 65%, 55%, or 45% from carbohydrate, respectively. Energy intake for each subject remained constant during the 12-week dietary intervention. A 7-day menu cycle was developed for each 4-week period using ProNutra metabolic feeding study software (ProNutra, Release 3.2, Viocare Technologies, Inc. Princeton, NJ, USA). Sources of protein followed those typically consumed by adults [25], including approximately 30% lean beef/pork (OMV) or soy/legume products (LOV), 20% dairy, 5% egg, 20% grains/breads/flours, and 25% other (*i.e.*, vegetables, fruits, nuts, and beverages). The 10%, 20%, and 30% of energy from protein was chosen because it spans the AMDR range for protein. Note the AMDR for protein is 10%–35% of energy intake during weight maintenance [24], but very few people habitually consume protein intakes at the upper limit of the range [24,26]. To ensure adequate micronutrient intakes subjects consumed one multivitamin/mineral supplement (Centrum Silver; Wyeth Consumer Healthcare, Richmond, VA, USA) and two calcium/vitamin D supplements (Citracal; Bayer Healthcare, LLC, Morristown, NJ, USA, 400 mg/day calcium; 500IU cholecalciferol per caplet) daily. *Ad libitum* water intake, salting and seasoning of food, and non-energy caffeine-containing beverages were allowed.

Diet Compliance: To encourage compliance, subjects were provided portioned quantities of selected foods (esp. beef/pork or soy/legume products) on days 1–24 of each 4-week period, and all foods and beverages on days 25–27 of each period. Subjects were counseled by a registered dietitian to follow their prescribed menus to achieve the desired macronutrient and energy intakes. A grocery list including brand names and a daily menu checklist with portion sizes for each food to purchase and consume were provided to the subjects. Throughout the study, subjects were encouraged to only consume the prescribed and provided foods and beverages and to report any non-compliance on the daily menu checklist.

Body Mass/Composition/Anthropometrics: Fasting-state body mass was measured twice weekly to document the effectiveness of and compliance with the energy-restricted diet. Fasting-state body mass (total mass—robe mass) was measured to the nearest 0.01 kg using a digital platform scale (model ES200L, Ohaus Corporation, Pine Brook, NJ, USA) and standing height without shoes was measured to the nearest 0.1 cm with a wall-mounted stadiometer. Body composition and waist and hip circumferences were measured at baseline on two separate days and on day 28 of each 4-week period. Body composition was determined, in duplicate, using a plethysmography system (BodPod, COSMED USA Inc.) which is sensitive to measuring small to moderate changes in body composition during weight loss [27]. Fat mass (FM), fat-free mass (FFM), and percent FM and FFM were calculated from body volume using the Siri equation [28]. Waist (natural and umbilical) and hip circumferences were measured in duplicate to the nearest millimeter using a spring-loaded tape measure.

Appetite Assessment: On days 25–27 of each 4-week period, subjects recorded their appetite (fullness, hunger, and desire to eat) upon awakening and hourly until bedtime using a 100-mm quasilogarithmic visual analog scale (*i.e.*, general labeled magnitude scale), with descriptors ranging from “barely detectable” to “strongest sensation imaginable of any kind” [29].

Fasting-State Resting Energy Expenditure (REE_f_): After a 10-h overnight fast and resting in a reclining position for 30 min, CO_2_ production and O_2_ consumption were measured by indirect calorimetry using a ventilated canopy (MedGraphics Cardiopulmonary Diagnostics Systems, Medgraphics Corporation, St. Paul, MN, USA). The Weir equation [30] was used to estimate REE_f_.

Blood Collection: Fasting blood samples were collected from an antecubital vein using venipuncture into serum- and plasma-separator tubes, centrifuged for 15 min at 4000 rpm and 4 °C, aliquoted into 1 mL microcentrifuge tubes, and stored at −80 °C until thawed for analysis.

Lipid-lipoprotein Profile: Fasting serum total cholesterol, HDL cholesterol (HDL-C), and triacylglycerol were analyzed by MidAmerica Clinical Laboratories using a photometric assay (Chemistry Immuno Analyser AU5700, Olympus, Center Valley, PA, USA). LDL cholesterol (LDL-C) was estimated using the following equation: LDL-C=total cholesterol − HDL-C − triacylglycerol/5 [31]. Apolipoprotein A1 (APO-A1) and apolipoprotein B (APO-B) were measured using a photometric assay (Cobas Integra 400, Roche Diagnostic Systems, Indianapolis, IN, USA).

Glucose and Insulin Analyses: Serum glucose was measured using a photometric assay (Cobas Integra 400; Roche Diagnostic Systems, Indianapolis, IN, USA). Serum insulin was measured using an electrochemiluminescence immunoassay (Elecsys 2010 Analyzer, Roche Diagnostic Systems, Indianapolis, IN, USA). Insulin resistance (HOMA-IR) was calculated using: (fasting glucose (mg/dL) × fasting insulin (µU/mL))/405 [32] and pancreatic β cell function (HOMA-β, %) was calculated using: (360 × fasting insulin (µU/mL))/(fasting glucose (mg/dL) − 65) [33].

Fasting Blood Pressure: After resting in a reclining position for 30 min, reclining and sitting systolic and diastolic blood pressures were measured in duplicate on the non-dominate arm using an automated sphygmomanometer.

Renal Response: Fasting serum blood urea nitrogen (BUN) and creatinine were measured using a photometric assay (Chemistry Immuno Analyser AU5700; Olympus, Center Valley, PA, USA) performed by MidAmerica Clinical Laboratories. BUN was used as a surrogate indicator of dietary protein intake during each period [34]. Glomerular filtration rate and creatinine clearance were estimated to assess renal function. Glomerular filtration rate was determined using the CKD-EPI (Chronic Kidney Disease Epidemiology Collaboration) equation where S_cr_ is serum creatinine (mg/dL): κ is 0.7 for females and 0.9 for males, α is −0.329 for females and −0.411 for males, min indicates the minimum of S_cr_/κ or 1, and max indicates the maximum of S_cr_/κ or 1: Glomerular filtration rate = 141 × min (S_cr_/κ,1)^α^ × max(S_cr_/κ,1)^−1.209^ × 0.993^Age^ × 1.018 (if female) × 1.159 (if black) [35]. Creatinine clearance rate (mL/min) was determined using the Cockcroft-Gault equation where S_cr_ is serum creatinine (mg/dL), BM is body mass in kg: creatinine clearance rate = [(140 − age) × BM/(S_cr_ × 72)] × 0.85 (if female) [36].

#### 2.2.3. Acute Feeding Component of Study (Day 28 of Each 4-Week Period)

Breakfast Meal: On day 28 of each 4-week period, subjects reported to the clinical laboratory after at least a 10-h overnight fast. Subjects were counseled to not consume any caffeine or to purposely exercise on the mornings of testing. Following fasting blood sampling, blood pressure, appetite, and REE_f_ measurements were made, subjects consumed a test meal providing 25% of their daily energy prescription (−750 kcal/day restriction) with protein quantity and source corresponding to the period-specific diet. The test meal consisted of portioned quantities of a breakfast sandwich containing English muffin, lean beef/pork (OMV) or soy breakfast patties (LOV) and cheese, and served with fruit and juice. Each subject was given 15 min to finish the test meal. Blood samples were collected and appetite questionnaires were completed at minutes 15, 25, 60, 85, 120, 180, and 240 post-meal. During this time, subjects were not allowed to read, watch TV, talk on a cell phone, work on a computer or eat or drink anything except for what was provided. After completion of the 4-h testing period, lunch was eaten on-site and pack-out foods for dinner were provided.

Postprandial Resting Energy Expenditure (REE_pp_): REE_pp_ was measured at 30–60, 90–120, 150–180, 210–240 min post-meal using the indirect calorimetry technique and equipment described above.

Plasma Free Amino Acids: For a subsample of subjects (5F OMV; 1M: 4F LOV) plasma free amino acids were quantified by the Agricultural Experimental Station Chemical Laboratories University of Missouri-Columbus [37]. The blood samples used were from time points fasting, 25, 60, 120, 180, and 240 min post-meal.

### 2.3. Data and Statistical Analyses

#### 2.3.1. Power Calculation

Due to the novelty of the current study objectives, the study was primarily powered to statistically confirm the differential effects of protein quantity on fullness, with sufficient subjects in the OMV and LOV groups separately. Other parameters, such as hunger, desire to eat, resting energy expenditures, and cardio-metabolic health indices were not considered when determining group sample size. As a result, these outcomes of interest were deemed secondary. Results from our previous research [3] showed that overweight and obese women who consumed a higher-protein diet (30% of energy, with 40% of total protein from pork) *versus* a lower protein diet (18% of energy and no meats consumed) during a 12-week period of energy restriction (–750 kcal/day) had a differential change in meal-related fullness rating of 18 ± 17 mm (mean ± SEMs, greater fullness for higher-protein group). Based on these results, *n* = 17 subjects from each group would be needed to detect a differential response with 80% power at α = 0.05.

#### 2.3.2. Data Analysis

Weight and body composition changes over time were analyzed using repeated measures ANOVA for OMV and LOV groups without regard to protein quantity to confirm the effectiveness of the weight loss diets. Repeated measures ANOVA was used to assess the main effects of protein source, quantity, and source by quantity interaction on appetitive, thermogenic, and cardio-metabolic outcomes. *Post hoc* pairwise comparisons were conducted using the Least Significant Difference method. Areas under the curve (AUC) for daily and postprandial appetite ratings were calculated using the trapezoid rule. AUCs for postprandial glucose, insulin, amino acids, and energy expenditure were calculated using the Wolever method [38]. The thermic effect of feeding was calculated by dividing the AUC of postprandial resting energy expenditure by the energy content of the meal. Daily appetitive responses (days 25–27 of each 4-week period) were each truncated to include a total of 14 time points (13-h period). Data are reported as Means ± SEMs and statistical significance was assigned at *p* < 0.05. Statistical analyses were performed using SPSS (version 22.0; IBM Corporation, Armonk, NY, USA).

## 3. Results

### 3.1. Subject Baseline Characteristics

Age, body mass, height, BMI, body composition (Table 1), lipid-lipoprotein, indices of fasting-state glucose homeostasis (fasting serum glucose and insulin, HOMA-IR, HOMA-β), blood pressures, REE_f_, and renal function indices (Table 2) were not different between the OMV and LOV groups at baseline.

**Table 1:** Baseline subject characteristics

Measurement	OMV (*n* = 17)	LOV(*n* = 17)
Gender	M = 5, F = 12	M = 6, F = 11
Age, year	51 ± 2	56 ± 4
Body mass, kg	87.0 ± 2.9	88.1 ± 2.9
Height, cm	167.5 ± 2.4	169.3 ± 2.3
Body mass index, kg/m^2^	31.0 ± 0.7	30.7 ± 0.6
Fat mass, kg	35.8 ± 2.0	34.8 ± 1.3
% Fat mass	41.5 ± 2.2	40.1 ± 1.0
Fat-free mass, kg	51.2 ± 3.0	53.3 ± 2.9
% Fat-free mass	58.5 ± 2.2	60.0 ± 1.6

Data are Means ± SEMs. There were no significant differences at baseline between groups.

The baseline estimated dietary energy requirement was not different between the OMV group (mean: 2497 ± 83, range 2043–3078 kcal/day) and LOV group (mean: 2490 ± 80, range 1957–3091 kcal/day), nor was the level of dietary restraint (9 ± 1 au for each group). The relative dietary energy restriction ranged from 24% to 37% among all subjects.

**Table 2:** Cardio-metabolic and renal responses of the omnivore (OMV) *vs.* lacto-ovo vegetarian (LOV) groups at baseline and at the end of each protein quantity-specific period

Measurement ^a^	Group	Baseline ^b^	10% Protein	20% Protein	30% Protein
**Fasting Lipid/Lipoprotein Status**					
Total cholesterol ^c^, mg/dL	OMV	186 ± 9	164 ± 5	151 ± 8	154 ± 8
	LOV	176 ± 6	149 ± 6	145 ± 6	143 ± 6
HDL-C, mg/dL	OMV	50 ± 3	44 ± 2	40 ± 3	42 ± 2
	LOV	53 ± 3	44 ± 2	44 ± 2	44 ± 2
LDL-C, mg/dL	OMV	112 ± 7	94 ± 5	89 ± 6	92 ± 5
	LOV	102 ± 6	83 ± 5	81 ± 4	82 ± 5
Triacylglycerol ^c^, mg/dL	OMV	135 ± 12	124 ± 9	113 ± 4	107 ± 9
	LOV	107 ± 10	115 ± 9	97 ± 9	99 ± 9
APO-A1 ^d^, mg/dL	OMV	145 ± 8	123 ± 5	122 ± 5	120 ± 4
	LOV	143 ± 7	121 ± 6	117 ± 4	118 ± 5
APO-B ^c^, mg/dL	OMV	91 ± 5	84 ± 4	81 ± 4	78 ± 3
	LOV	83 ± 4	77 ± 4	71 ± 3	70 ± 3
**Glycemic Status**					
Fasting glucose, mg/dL	OMV	86 ± 3	91 ± 2	90 ± 2	89 ± 2
	LOV	89 ± 2	92 ± 2	90 ± 1	91 ± 2
Fasting insulin, µU/mL	OMV	11.6 ± 1.6	8.3 ± 1.2	7.6 ± 0.8	10.1 ± 2.4
	LOV	9.7 ± 1.3	6.9 ± 0.8	6.2 ± 0.9	6.2 ± 0.7
HOMA-IR	OMV	2.54 ± 0.42	1.90 ± 0.29	1.73 ± 0.19	2.38 ± 0.68
	LOV	2.14 ± 0.29	1.59 ± 0.19	1.36 ± 0.20	1.42 ± 0.18
HOMA-β, %	OMV	144.9 ± 30.8	104.9 ± 11.9	98.9 ± 7.3	124.9 ± 25.2
	LOV	150.4 ± 25.0	87.5 ± 9.3	87.5 ± 13.3	88.8 ± 8.6
Glucose AUC ^e^, mg/dL × 240 min	OMV		1557 ± 282	1559 ± 278	2164 ± 288
	LOV		2182 ± 274	2156 ± 270	2245 ± 279
Insulin AUC ^ef^, µU/mL × 240 min	OMV		3964 ± 451	3367 ± 411	4565 ± 639
	LOV		4133 ± 365	3891 ± 443	4675 ± 637
**Blood Pressure Status ^g^**					
Reclining systolic BP, mm Hg	OMV	122 ± 2	116 ± 3	116 ± 3	112 ± 2
	LOV	123 ± 3	117 ± 3	118 ± 4	115 ± 3
Reclining Diastolic BP, mm Hg	OMV	81 ± 2	77 ± 2	76 ± 2	73 ± 1
	LOV	78 ± 2	75 ± 1	76 ± 2	75 ± 2
Sitting Systolic BP, mm Hg	OMV	116 ± 3	111 ± 3	112 ± 2	108 ± 2
	LOV	120 ± 4	115 ± 3	112 ± 4	110 ± 3
Sitting Diastolic BP, mm Hg	OMV	74 ± 2	70 ± 2	69 ± 2	67 ± 1
	LOV	74 ± 2	71 ± 2	70 ± 2	69 ± 2
**Fasting Renal Status ^g^**					
Serum Creatinine, mg/dL	OMV	0.90 ± 0.05	0.86 ± 0.04	0.90 ± 0.04	0.86 ± 0.05
	LOV	0.92 ± 0.04	0.83 ± 0.04	0.82 ± 0.03	0.82 ± 0.03
Creatinine clearance rate, mL/min	OMV	111 ± 6	108 ± 7	103 ± 6	110 ± 8
	LOV	104 ± 6	107 ± 7	107 ± 6	106 ± 6
Glomerular filtration rate, mL/min	OMV	83 ± 4	86 ± 4	83 ± 4	85 ± 4
	LOV	80 ± 4	88 ± 4	88 ± 4	87 ± 4
**Resting Energy Expenditure**					
REE_f_, kcal/kg/h ^h^	OMV	0.89 ± 0.04	0.90 ± 0.04	0.90 ± 0.04	0.87 ± 0.04
	LOV	0.92 ± 0.03	0.86 ± 0.05	0.90 ± 0.04	0.98 ± 0.04
REE_pp_ AUC ^e^, kcal/kg/h × 4 h	OMV		0.47 ± 0.09	0.40 ± 0.06	0.58 ± 0.11
	LOV		0.66 ± 0.01	0.58 ± 0.12	0.48 ± 0.10
Thermic effect of feeding ^i^ (%)	OMV		8.9 ± 1.6	7.7 ± 1.3	11.0 ± 2.1
	LOV		12.7 ± 2.0	11.1 ± 2.4	9.1 ± 1.8

^a^ Data are Means ± SEMs; ^b^ No differences at baseline between groups; ^c^ There was a main effect of protein quantity (*p* < 0.05). *Post-hoc*: 10% > 20% and 30% protein (*p* < 0.05); ^d^ LOV M = 6, F = 10 for 30% protein; ^e^ Area under the curve was calculated to represent the postprandial response to consuming the acute test breakfast; ^f^ There was a main effect of protein quantity (*p* < 0.05). *Post-hoc*: 20% < 30% (*p* < 0.05); ^g^ LOV M = 5, F = 11 for 30% protein; ^h^ There was a protein source-by-quantity interaction (*p* < 0.05). No main effects of protein source or quantity. *Post-hoc*: LOV 30% > OMV 30% protein, (*p* < 0.05); ^i^ The thermic effect of feeding was expressed as: REEpp AUC (kcal) divided by the calorie of the test breakfast.

### 3.2. Longitudinal Response to the Dietary Intervention

#### 3.2.1. Compliance

**Figure 3:**
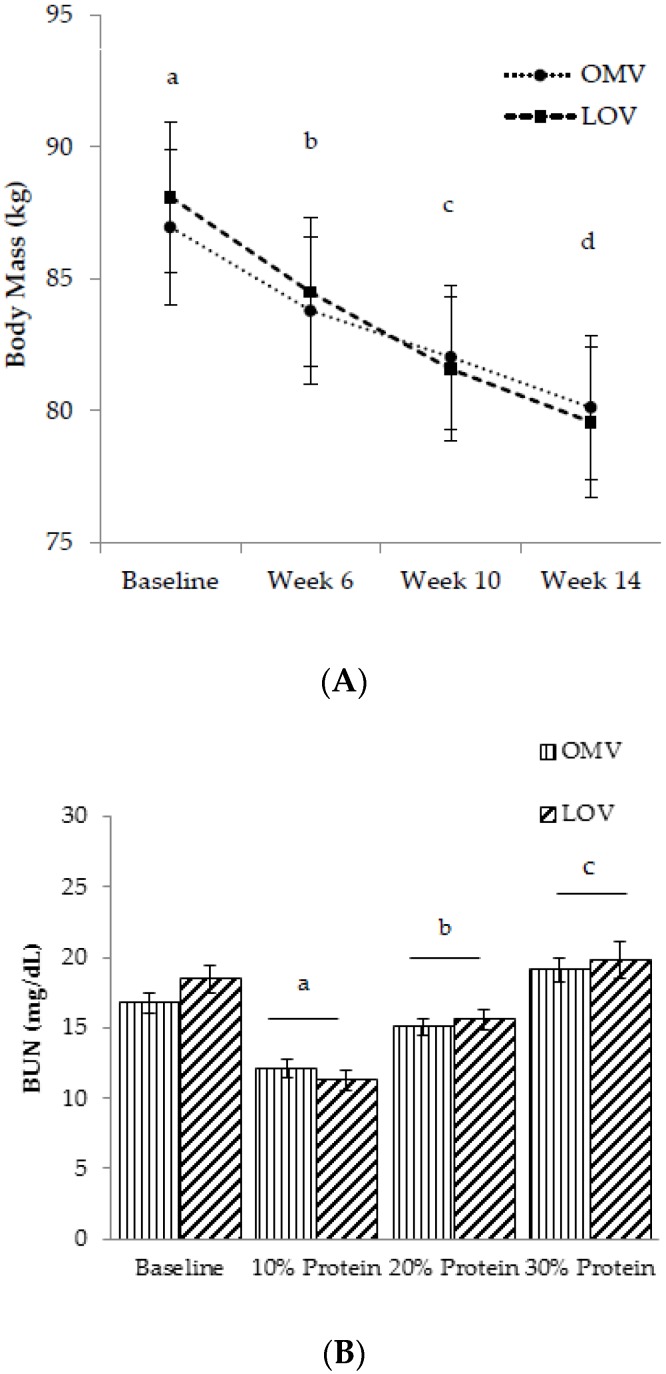
(**A**) Body mass status overtime; and (**B**) fasting blood urea nitrogen (BUN) at the end of each 4-week period. ^a,b,c,d^: Values with different letters differ significantly, *p* < 0.001.

Compliance to the study diets was confirmed by both body mass loss (Figure 3A) and fasting BUN (Figure 3B) at the end of each 4-week period. BUN concentration, a reflection of dietary protein consumption, was not different between the OMV (17 ± 1 mg/dL) and LOV (18 ± 1 mg/dL) groups at baseline. At the end of the 10%, 20%, and 30% protein intake periods, BUN progressively increased with increasing protein quantity (*p* < 0.001), independent of protein source. In addition, reductions of body mass over the 12-week intervention further suggested dietary compliance. Subjects lost an average of 7.7 ± 0.4 kg body mass during the 12-week intervention. Additional data on changes in BMI, fat mass, % fat mass, fat-free mass, % fat-free mass, and waist to hip ratio over time are reported in Supplementary Table S1.

#### 3.2.2. Appetite

**Figure 4:**
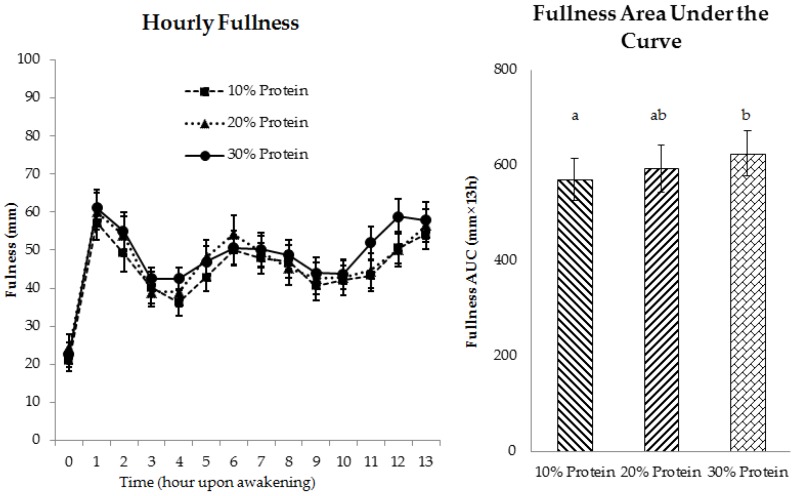
Daily fullness rating at the end of each 4-week period (days 25–27) independent of protein source. ^a,b^: Values with different letters differ significantly, *p* = 0.03.

Daily appetitive responses, including fullness, hunger, and desire to eat, at the end of the 4-week energy-restricted periods were not affected by the source of protein (Figure 4 and Figure S1). Daily composite fullness AUC was higher when 30% protein was consumed, compared to 10% protein (*p* = 0.03), with the 20% protein fullness response intermediate to the higher and lower protein intakes (Figure 4). Protein quantity did not affect daily composite hunger or desire to eat (Figure S1). Hourly appetite ratings (shown in Figure 4 and Figure S1) were not compared statistically because the subjects self-chose when to eat during the day.

#### 3.2.3. Fasting-State Resting Energy Expenditure (REE_f_)

REE_f_ was not influenced by protein quantity in the OMV group, but tended to progressively increase with higher protein intake in the LOV group (protein source by quantity interaction: *p <* 0.05, Table 2). *Post-hoc* analysis showed that REE_f_ was greater when 30% protein from LOV was consumed *vs.* 30% protein from OMV (*p <* 0.05).

#### 3.2.4. Metabolic Health

During energy restriction, the source and quantity of protein did not affect HDL-C, LDL-C, APO-A1, fasting glucose, fasting insulin, HOMA-IR, HOMA-β, blood pressure parameters, or renal status (creatinine concentration, creatinine clearance rate, and glomerular filtration rate) (Table 2). Independent of protein source, lower fasting concentrations of total cholesterol, triacylglycerol, and APO-B were observed at the end of the 20% and 30% protein periods compared to the 10% protein period (*p <* 0.05).

### 3.3. Postprandial Responses to the Breakfast Test Meal (Day 28 of Each 4-Week Controlled Feeding Period)

#### 3.3.1. Amino Acid Profiles

**Figure 5:**
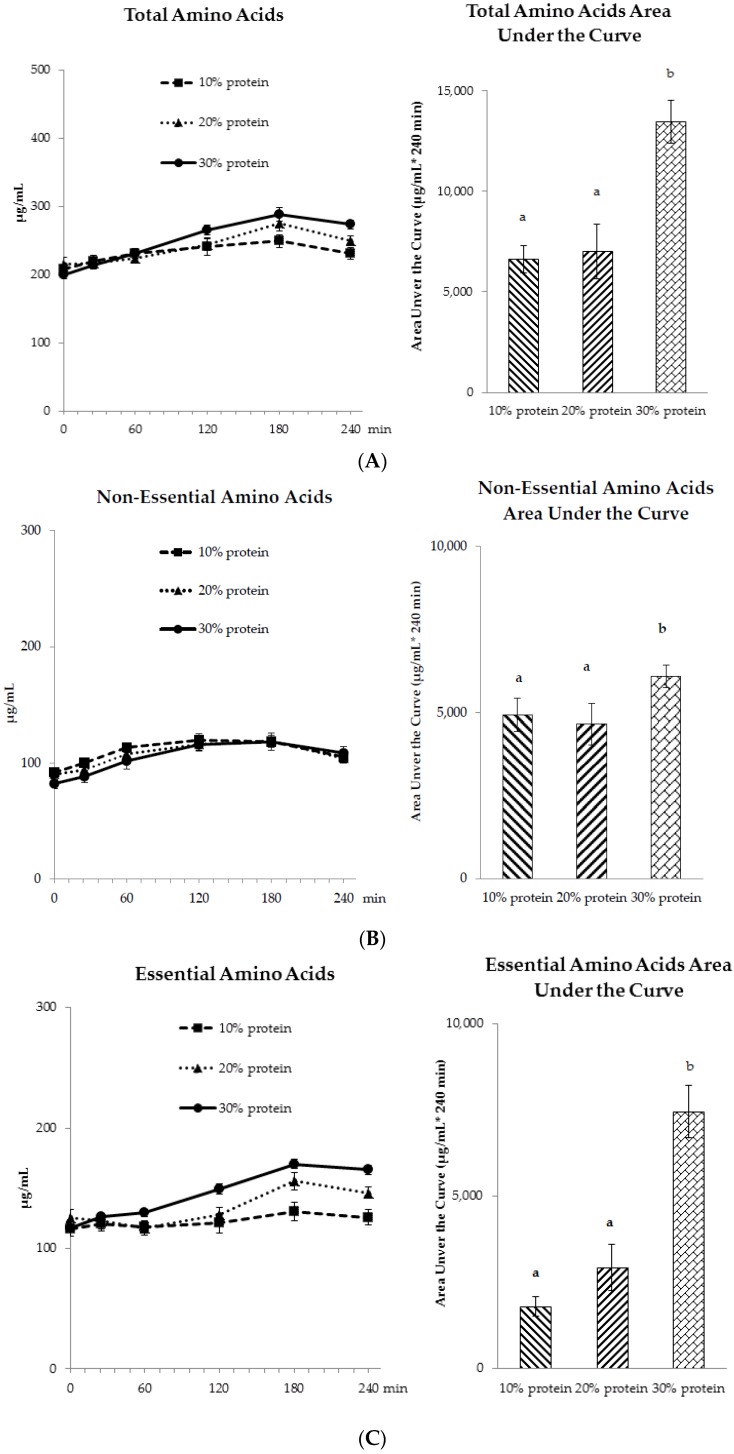
Postprandial plasma free (**A**) total amino acids; (**B**) non-essential amino acids; and (**C**) essential amino acids profiles at the end of each 4-week period (day 28). ^a,b^: No effect of protein source was observed; values with different letters differ significantly, *p* < 0.001.

Post-meal plasma free amino acid concentrations reached peaks approximately 3 h after meal ingestion (Figure 5A–C). The areas under the curve for total, essential, and non-essential amino acids did not differ between protein sources. The 30% protein breakfast meal had higher total amino acids, non-essential amino acids, and essential amino acids AUCs compared to the 10% and 20% protein breakfast meals (*p* < 0.001).

#### 3.3.2. Appetite

**Figure 6:**
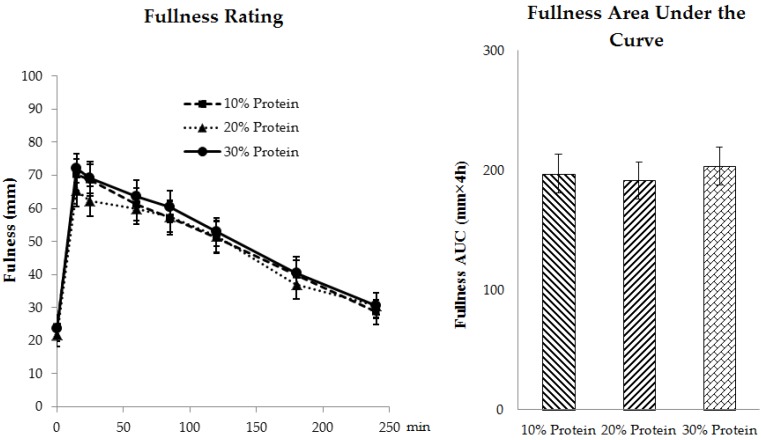
Postprandial ratings of fullness at the end of each 4-week period (day 28) independent of protein source

Neither protein source nor quantity affected the postprandial appetite ratings as reflected by the AUCs for fullness (Figure 6), hunger or desire to eat over the 240-min testing period (Figure S2).

#### 3.3.3. Postprandial Energy Expenditures (REE_pp_)

Neither protein source nor quantity affected the 4-h postprandial increases in resting energy expenditure when expressed as AUCs or percentages of total energy consumed (thermic effect of feeding, %) (Table 2).

#### 3.3.4. Postprandial Glycemic and Insulinemic Responses

Neither protein source nor quantity influenced glucose AUCs through the 4-h postprandial period. Independent of source, insulin AUC was elevated after consuming breakfast meals containing 30% protein compared to 20% protein (*p* = 0.02) (Table 2).

## 4. Discussion

We systematically investigated the effect of protein source and quantity across the AMDR on appetite and energy expenditures among individuals who consumed the energy-restricted diets for 4 weeks, which is sufficient time for the subjects to become acclimated to the protein intake [39]. Multiple measures reflected that subjects were compliant to the study diets: the body mass loss and blood urea nitrogen data indicated that subjects were consuming the energy-restricted diets and the prescribed protein quantity, respectively. The amino acid responses following the acute meal ingestion further reflected that the meals were different in protein quantity. In addition, we investigated appetitive responses under controlled-feeding within free-living and in-lab environments. The free-living environment allowed us to assess perceived appetitive responses among subjects without being confounded by compensatory eating behaviors or changes in daily activity patterns. The in-lab environment provided a more controlled setting which allowed us to assess the effect of protein source and quantity in isolation without being confounded by other environmental, social, and behavioral factors that may be present in a free-living condition [40]. Furthermore, we used a mixed meal and whole food approach, where the study diets contained beef/pork or soy/legume as the predominant protein sources (30% of total protein intake), as well as proteins from various sources commonly consumed by Americans [26]. Such diets are comparable to an individual’s protein intake patterns, thus the results can be readily applied by the general populous who are dieting.

We acknowledge that the study had potential limitations. First, no washout periods were included between each 4-week period with different protein quantities. This was due to the logistics of recruiting and retaining subjects for a weight loss study. To minimize the effect of diet order on the study outcomes, we randomly assigned the order of protein intake periods in a balanced manner. In addition, subjects’ appetitive responses were measured using visual analog ratings only. We did not measure subsequent food intake following the in-lab breakfast test, thus it is unknown whether the perceived ratings for appetite would translate into ingestive behaviors. In addition, postprandial responses of hormones related to appetite were not measured, which would provide more information about protein intake and appetite control.

### 4.1. Appetite

Despite a vast body of evidence supporting the beneficial effects of *ad libitum* high protein diets on appetite [41], a paucity of studies exist that evaluated appetite after subjects had habitually consumed an ER diet with the specified amount of protein. Some diet-related intervention studies found greater satiety/fullness ratings [3,10,12], but not others [9,11] when higher protein was consumed during ER-induced weight loss. The studies that reported hunger [3,12] and desire to eat [3,9] did not support an effect of protein quantity on these indices. Most of these studies assessed daily or weekly appetite from a general diet satisfaction survey [9,10,11,12], instead of hourly visual analog scale ratings over multiple days [3]. Such inconsistencies in methodologies caused challenges for interpreting previous research. The current study adds to the literature that dietary protein intake across the AMDR does not influence daily or postprandial hunger or desire to eat, but supports that 30% protein diet results in moderately higher daily composite fullness rating, compared to the 10% protein diet.

The results from several studies that also used a whole food approach are consistent with our finding that protein source does not affect appetite ratings. Neacsu *et al.* compared vegetarian and meat-based high protein diets (30% of energy from protein) on appetite after a 2-week period of energy restriction and found no effects of protein source on appetite ratings [42]. Other research that compared a mixed meal with beef *vs.* soy [43] or beans [20] showed that the protein source did not affect appetite and subsequent food intake. The current study expanded these previous studies of protein source on appetitive responses and systematically compared protein intake across the AMDR (10%, 20%, and 30% protein) within the context of energy restriction. Our data support these observations and suggest that upon 4-week acclimation to the study diets, subjects’ appetitive responses do not differ among diets with beef/pork or soy/legume products as the predominant protein sources.

### 4.2. Fasting Energy Expenditure

A recent meta-analysis of energy-restriction-induced weight loss studies (*n* = 4) showed beneficial effects of high protein on preserving resting energy expenditure during weight loss [4]. When looking closer at the included studies, the result may be driven by the two studies that compared high protein (45%) *vs.* lower protein (12%) [44,45]. When comparing moderately high protein intake to normal protein intake during weight loss (32% *vs.* 15%, 27% *vs.* 16%), the other two studies showed that resting energy expenditure was not differentially influenced [10,46]. Our findings generally support that protein intake within the AMDR does not affect fasting resting energy expenditure. However, caution is warranted with this interpretation because of the statistically significant protein source by quantity interaction. While the results suggest that a 30% protein intake from a lacto-ovo vegetarian diet resulted in higher REE_f_ compared to an omnivorous diet, this observation should be confirmed by further research.

### 4.3. Thermic Effect of Feeding

Previous acute feeding studies showed that without prior weight loss or acclimation to protein intake, high protein meals lead to slight, but significantly higher TEF compared to lower protein meals [14,15]. However, Luscombe *et al.* found that although at baseline TEF was higher with meals consisting 27% *vs.* 16% protein, this discrepancy disappeared at the end of the 16-week weight loss/maintenance intervention among adults with type 2 diabetes [46]. We observed that upon acclimation to protein intake and weight loss, the TEF of meals with varying protein quantities did not differ. These results suggest that acclimation may be an important determinant of the thermic effect of feeding with different amounts of protein. With regards to protein source, a previous acute study comparing meals with pork *vs.* soy proteins suggested that protein source affected the TEF over 24 h using a respiratory chamber (pork > soy) [19], but our results do not support this observation. It is unknown whether the inconsistent findings were due to the acclimation of the weight loss/protein intake or differences in methodologies (e.g., respiratory chamber *vs.* metabolic cart; 24 h *vs.* 4 h indirect calorimetry). Previous studies suggested that the elevation of resting energy expenditure after consuming meals last more than 6 h [47,48]. The 4-h time frame for TEF measurement may not be sufficiently long to detect differences in TEF for the test meals.

### 4.4. Cardio-Metabolic Indices

Our study showed that the metabolic indices were not affected by the protein source and quantity at the end of each 4-week period, with the exception of lower cholesterol, triglycerides, and APO-B when 20% or 30% protein was consumed compared to 10% protein. This is consistent with the aforementioned meta-analysis which observed a modest reduction in triglycerides with higher protein intake [4]. This improvement in triglycerides was primarily due to reduced carbohydrate intake. In addition, we observed that the postprandial glycemic responses were not influenced by protein quantity or source, although the 30% protein breakfast resulted in higher insulin responses than 20% protein. This result is in agreement with the observed insulinotropic properties of proteins [49,50].

## 5. Conclusions

The results of this randomized, crossover, diet-controlled study indicate that indices of appetite (daily and postprandial) and resting energy expenditure (fasting and postprandial) are minimally influenced by dietary protein intakes that span the AMDR among adults who are overweight/obese after they become acclimated to these energy-restricted diets. The use of beef/pork *vs.* soy/legumes as the predominant sources of protein does not affect these findings.

## Supplementary Materials

Supplementary materials are available online at http://www.mdpi.com/2072-6643/ 8/2/63/s1.
