# Supplementary material for: Effects of Dietary Protein Source and Quantity during Weight Loss on Appetite, Energy Expenditure, and Cardio-Metabolic Responses
Source: Nutrients. 2016 Jan 26;8(2):63. doi: 10.3390/nu8020063 (PMC4772027; doi:10.3390/nu8020063)
Supplement: Supplementary file 1 [file nutrients-08-00063-s001.docx]

Supplementary Materials: Dietary Protein Source and Quantity during Weight Loss do not Impact, Appetite, Energy Expenditure, or Cardio-Metabolic Responses

Jia Li, Cheryl L. H. Armstrong and Wayne W. Campbell

**Table S1.** Changes in weight status, body composition, and other anthropometric measurements among subjects in OMV and LOV groups throughout the study.

| **Measurement ^a^** | **Group** | **Baseline** | **Weeks 6** | **Weeks 10** | **Weeks 14** | **Change ^b^ (Post − Baseline)** |
| --- | --- | --- | --- | --- | --- | --- |
| Body mass ^c^, kg | OMV | 87.0 ± 2.9 | 83.8 ± 2.8 | 82.0 ± 2.7 | 80.1 ± 2.9 | −6.8 ± 0.6 |
|  | LOV | 88.1 ± 2.9 | 84.5 ± 2.8 | 81.6 ± 2.7 | 79.6 ± 2.7 | −8.5 ± 0.6 |
| Body mass index ^c^, kg/m^2^ | OMV | 31.0 ± 0.7 | 29.8 ± 0.6 | 29.2 ± 0.7 | 28.5 ± 0.7 | −2.4 ± 0.2 |
|  | LOV | 30.7 ± 0.6 | 29.4 ± 0.6 | 28.4 ± 0.6 | 27.7 ± 0.6 | −3.0 ± 0.2 |
| Fat mass ^c^, kg | OMV | 35.8 ± 2.0 | 33.2 ± 1.8 | 31.8 ±2.1 | 29.7 ± 2.1 | −6.1 ± 0.6 |
|  | LOV | 34.8 ± 1.3 | 32.5 ± 1.4 | 29.6 ± 1.6 | 28.1 ± 1.6 | −6.8 ± 0.7 |
| % fat mass ^c^ | OMV | 41.5 ± 2.2 | 40.0 ± 2.1 | 39.0 ± 2.4 | 37.2 ± 2.3 | −4.3 ± 0.6 |
|  | LOV | 40.1 ± 1.0 | 38.8 ± 1.7 | 36.6 ±1.9 | 35.5 ± 1.8 | −4.5 ± 0.7 |
| Fat-free mass ^d^, kg | OMV | 51.2 ± 3.0 | 50.6 ± 2.8 | 50.2 ± 2.9 | 50.5 ± 2.9 | −0.7 ± 0.4 |
|  | LOV | 53.3 ± 2.9 | 52.0 ± 2.7 | 52.0 ± 2.7 | 51.5 ± 2.7 | −1.8 ± 0.5 |
| % fat-free Mass ^e^ | OMV | 58.5 ± 2.2 | 60.0 ± 2.1 | 61.0 ± 2.4 | 62.8 ± 2.3 | 4.3 ± 0.6 |
|  | LOV | 60.0 ± 1.6 | 61.2 ± 1.7 | 63.4 ± 1.9 | 64.5 ± 1.8 | 4.5 ± 0.7 |
| Natural waist circumference ^c,f^, mm | OMV | 987 ± 25 | 956 ± 22 | 923 ± 24 | 911 ± 22 | −76 ± 10 |
|  | LOV | 996 ± 23 | 965 ± 23 | 936 ± 22 | 922 ± 24 | −68 ± 8 |
| Umbilical waist circumference ^d,f^, mm | OMV | 1047 ± 20 | 1017 ± 23 | 985 ± 24 | 964 ± 21 | −83 ± 11 |
|  | LOV | 1057 ± 16 | 1033 ± 16 | 1001 ± 16 | 985 ± 16 | −66 ± 8 |
| Hip circumference ^g,h^, mm | OMV | 1166 ± 49 | 1151 ± 53 | 1112 ± 53 | 1081 ± 45 | −64 ± 21 |
|  | LOV | 1120 ± 13 | 1081 ± 16 | 1054 ± 16 | 1032 ± 17 | −78 ± 12 |
| Waist to hip ratio | OMV | 0.86 ± 0.03 | 0.85 ± 0.03 | 0.85 ± 0.03 | 0.86 ± 0.03 | −0.017 ± 0.017 |
|  | LOV | 0.89 ± 0.03 | 0.89 ± 0.02 | 0.89 ± 0.03 | 0.90 ± 0.03 | 0.002 ± 0.009 |

^a^ Mean ± SEMs. There were no significant differences at baseline between groups; ^b^ Weeks 14 − Baseline. There were no significant differences in the change over time between groups; ^c^ Independent of protein source there was a main effect of time (*p* < 0.001). *Post-hoc*: Baseline > Weeks 6 > Weeks 10 > Weeks 14 (*p* < 0.001 for all); ^d^ Independent of protein source there was a main effect of time (*p* < 0.001). Post-hoc: Baseline > Weeks 6, 10 and 14 (*p* < 0.01 for all); ^e^ Independent of protein source there was a main effect of time (*p* < 0.001). *Post-hoc*: Baseline < Weeks 6 < Weeks 10 < Weeks 14 (*p* < 0.001 for all); ^f^ OMV: M = 5, F = 11 at Week 6; LOV: M = 5, F = 11 at Baseline; ^g^ Independent of protein source there was a main effect of time (*p* < 0.001). *Post-hoc*: Baseline > Weeks 6 > Weeks 10 (*p* < 0.05 for all); ^h^ OMV: M = 5, F = 10 at Weeks 6 and 14; LOV: M = 5, F = 11 at Baseline.


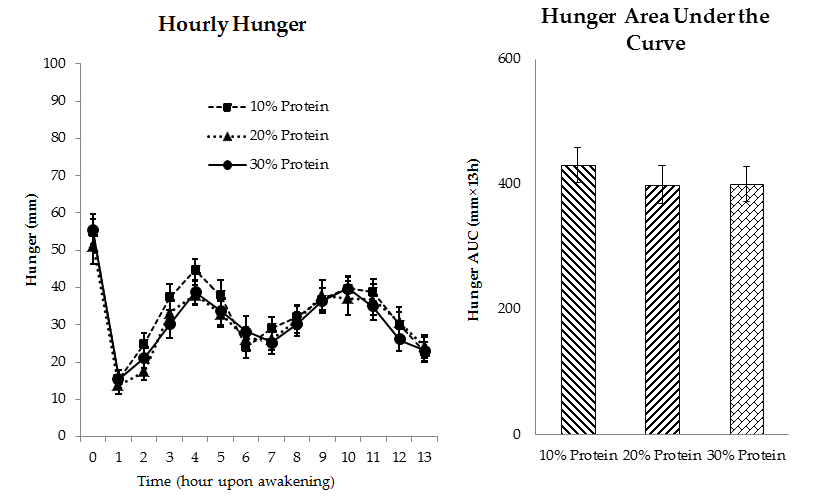


(A)


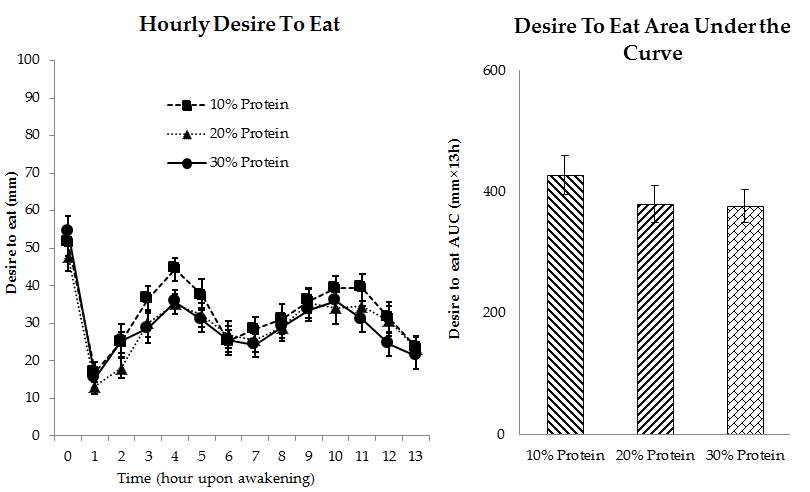


(B)

**Figure S1.** Daily ratings of (**A**) hunger and (**B**) desire to eat at the end of each 4-weeks (days 25–27) period independent of protein source.


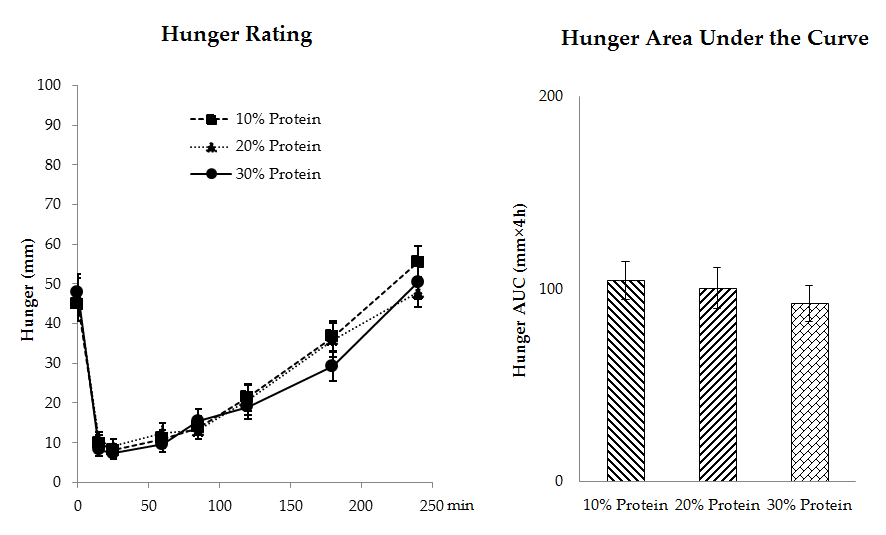


(A)


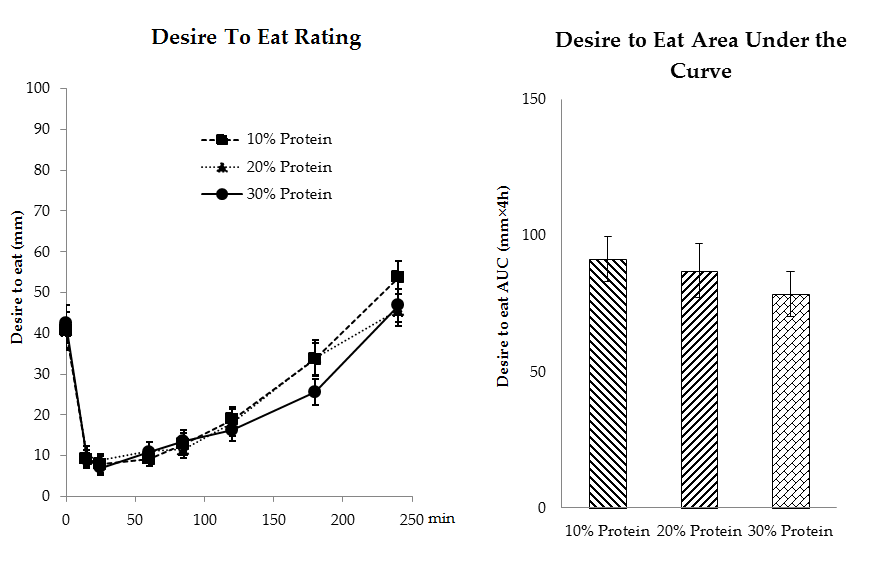


(B)

**Figure S2.** Postprandial ratings of (**A**) hunger and (**B**) desire to eat at the end of each 4-week (day 28) period independent of protein source.
